# De-Escalated Therapy and Early Treatment of Recurrences in HPV-Associated Head and Neck Cancer: The Potential for Biomarkers to Revolutionize Personalized Therapy

**DOI:** 10.3390/v16040536

**Published:** 2024-03-29

**Authors:** Wendell G. Yarbrough, Travis P. Schrank, Barbara A. Burtness, Natalia Issaeva

**Affiliations:** 1 Department of Otolaryngology/Head and Neck Surgery, UNC School of Medicine, Chapel Hill, NC 27599, USA; travis_schrank@med.unc.edu (T.P.S.); natalia.isaeva@med.unc.edu (N.I.); 2 Department of Pathology and Lab Medicine, UNC School of Medicine, Chapel Hill, NC 27599, USA; 3 Lineberger Comprehensive Cancer Center, UNC School of Medicine, Chapel Hill, NC 27599, USA; 4 Department of Medicine, Medical Oncology, Yale School of Medicine, New Haven, CT 06510, USA; barbara.burtness@yale.edu; 5 Yale Cancer Center, Yale School of Medicine, New Haven, CT 06510, USA

**Keywords:** HPV, head and neck cancer, head and neck squamous cell carcinoma, HNSCC, biomarkers, cancer subtypes, therapy deintensification, therapy de-escalation, liquid biopsy

## Abstract

Human papillomavirus-associated (HPV+) head and neck squamous cell carcinoma (HNSCC) is the most common HPV-associated cancer in the United States, with a rapid increase in incidence over the last two decades. The burden of HPV+ HNSCC is likely to continue to rise, and given the long latency between infection and the development of HPV+ HNSCC, it is estimated that the effect of the HPV vaccine will not be reflected in HNSCC prevalence until 2060. Efforts have begun to decrease morbidity of standard therapies for this disease, and its improved characterization is being leveraged to identify and target molecular vulnerabilities. Companion biomarkers for new therapies will identify responsive tumors. A more basic understanding of two mechanisms of HPV carcinogenesis in the head and neck has identified subtypes of HPV+ HNSCC that correlate with different carcinogenic programs and that identify tumors with good or poor prognosis. Current development of biomarkers that reliably identify these two subtypes, as well as biomarkers that can detect recurrent disease at an earlier time, will have immediate clinical application.

## 1. Introduction

For decades, patients with head and neck cancer suffered poor survival rates, prompting physicians and researchers to seek more effective therapies. The focus of many trials was to increase treatment intensity, balanced by the need to limit short- and long-term toxicities; nonetheless, standard treatment migrated toward the most aggressive tolerable regimens. Combining therapeutic modalities, adding targeted therapies, accelerating radiation fractionation, improving surgical techniques, and adding, changing, or escalating dose of chemotherapeutic agents either failed to improve or incrementally improved survival. More rapid advancement in survival was limited by a lack of molecular understanding of appropriate therapeutic targets, late effects of treatment toxicity, lack of effective agents, and/or inability to match patients to their most effective therapy. Major shifts to this paradigm for HPV-associated HNSCC are emerging, driven by development of antibodies targeting immune checkpoints and molecular characterization of tumors and stroma, leading to improved understanding of the distinct biology and vulnerabilities of these tumors.

The concept of HPV as an etiologic agent causing oropharyngeal cancer was met with skepticism in the 1980s and 1990s, but by the turn of the millennium, data supported the concept, leading to its widespread acceptance. Since then, the incidence of HPV+ head and neck cancer has continued to rapidly increase, and it now represents the majority of oropharyngeal cancers and is more frequent than uterine cervical cancer in the United States [1]. When last reported in 2016, 13,500 HPV+ HNSCC cancers were estimated yearly in the United States. Based on 2016 cancer statistics, this represents 22% of cancers of the upper aerodigestive tract and 82% of oropharyngeal cancers [1,2].

HPV+ HNSCC were found to be molecularly and clinically distinct from the HPV-negative tumors. While classic tobacco-driven HNSCC occurs in all sites of the upper aerodigestive tract, HPV+ tumors primarily occur in the oropharynx. Marked differences in gene expression, methylation, mutation profile, and mutagenic drivers distinguish these tumors [3]. Association of HPV+ HNSCC with improved prognosis and treatment response, younger patient age, and longer expected lifespan relative to HPV-negative disease spurred clinicians and researchers to question whether maximally aggressive treatments were in the best interest of patients or were needed to effectively treat these tumors. Although aggressive therapy remains the standard for HPV+ HNSCC, institutional and national clinical trials have studied a variety of de-escalation protocols with patient selection reliant on tumor stage, smoking history, response of tumors to induction therapy, or pathological characteristics after surgery. While many of these trials have exciting preliminary results, patient selection has remained a significant challenge to avoid over- or undertreatment.

As the head and neck research community strives to develop new or improved therapies, studies of HPV-associated HNSCC are continuously advancing our understanding of this disease. Genomic defects found in a subset of HPV-associated HNSCC provided some of the first insights distinguishing two subtypes of HNSCC, one associated with good and one associated with poor survival. By further defining these subtypes, a new mechanism of HPV carcinogenesis and potential therapeutic targets have been revealed.

In this article, we focus on HPV-associated head and neck squamous cell carcinoma (HNSCC) and the clinical impact of biomarkers driven by increased knowledge of its unique molecular characteristics, improved understanding of HPV carcinogenesis, and technological advances that can be leveraged to improve patient care and outcomes.

## 2. History of Therapeutic Escalation for HNSCC

Starting in the 1980s, curative therapy for head and neck cancers began to incorporate chemotherapy in the standard definitive and postoperative radiation regimens. While single-modality therapy for early-stage tumors had high rates of cure and acceptable morbidity with either surgery or radiation, unacceptably high recurrence rates and poor overall survival for locoregionally advanced head and neck cancer led to studies exploring treatment intensification with induction, concurrent chemotherapy, or altered fractionation radiation schemes. Determining the best strategy based on chemotherapy combinations, dose, and timing relative to surgery or radiation has dominated trials over the last several decades, ultimately, with intensification limited by tissue toxicity and patient morbidity.

Before the widespread availability of free flap reconstruction techniques, surgical morbidity or tumor extent rendered many tumors unresectable, and cure or control of these unresectable locoregionally advanced tumors with radiation was infrequent. The Head and Neck Intergroup study of unresectable disease compared standard radiation to either concurrent radiation with cisplatin or radiation plus 5FU and cisplatin with an interim analysis for resectability after delivery of approximately half of the radiation dose. Overall survival (OS) and disease-free survival (DFS) favored either chemotherapy arm over the standard single-modality radiation arm, but with increased toxicity attributable to chemotherapy [4]. DFS and OS also favored the cisplatin/radiation arm. The benefit of concomitant chemoradiation over radiation alone was confirmed in a meta-analysis of patients enrolled in 50 trials [5]. Pooled analysis demonstrated a 0.81 hazard of death, with an absolute survival benefit for addition of chemotherapy of 6.5% at 5 years. Prior meta-analysis showed no survival benefit for concomitant chemoradiation in trials conducted in larynx cancer [6], a setting with a high success rate for surgical salvage.

An alternate strategy to improve outcome in locally advanced head and neck cancer is altered radiation fractionation, tested in various schemas in 15 randomized trials. Meta-analysis of these trials demonstrated that overall survival was significantly improved by altered fractionation (2% at 5 years) and hyperfractionation (8% at 5 years) [7]. There was also a benefit of altered or hyperfractionation to local control, although this effect was less apparent for nodal control. The relative merit of these approaches has been evaluated in only a few trials, most notably in the GORTEC 99-02 trial. Here, patients were randomized among chemoradiation to 70 Gy in 7 weeks with three cycles of concomitant carboplatin-fluorouracil over 4 days, accelerated chemoradiation to 70 Gy in 6 weeks with two cycles of carboplatin-fluorouracil over 5 days, or very accelerated radiation to 64.8 Gy in bid 1.8 Gy fractions over 3.5 weeks [8]. The trial demonstrated that concurrent chemoradiation improved PFS compared to very accelerated radiation with a hazard ratio of 0.82, *p* = 0.041, corresponding to a 5.4% improvement in 3-year PFS. Additionally, altered fractionation increased acute toxicity. An additional question is whether chemotherapy added to an altered radiation fractionation schedule would lead to additive benefit. In GORTEC 99-02, accelerated chemoradiation offered no benefit over conventional chemoradiation. The RTOG 0129 trial compared 70 Gy radiation given concurrently with three cycles of cisplatin 100 mg/m^2^ over 7 weeks to 72 Gy radiation given concurrently with two cycles of cisplatin 100 mg/m^2^ over 6 weeks, with no differences in median or 8-year survival [9]. Thus, conventionally fractionated radiation with concurrent chemotherapy has remained the standard of care.

For advanced laryngeal cancer, a cooperative group three-arm trial compared induction chemotherapy followed by radiation to concurrent cisplatin and radiation to radiation alone. Early results favored concurrent cisplatin with radiation for both larynx preservation and DFS [10] and further established concurrent cisplatin/radiation as the standard for head and neck cancer. However, long-term analysis of this trial revealed a numerical reduction in 5- and 10-year survival for patients treated with concurrent chemoradiation compared to patients treated on the induction chemotherapy arm [11]. Ten years after therapy, patients treated with induction chemotherapy had an absolute 10% higher overall survival (39% vs. 28%) compared to patients treated with concurrent chemoradiation. Late deaths in this trial were not related to locoregional or distant recurrences, suggesting that treatment toxicity for patients treated with concurrent cisplatin and radiation may increase noncancer mortality within 10 years of therapy.

The role of induction chemotherapy has also been evaluated in numerous randomized trials. Meta-analysis suggested that chemotherapy prior to radiation improved outcome, but with a lesser benefit than shown in trials of concurrent chemoradiation [6]. Randomized trials adding induction chemotherapy before chemoradiation have demonstrated survival benefit in virally-associated nasopharynx cancer [12], but not in locally advanced non-nasopharyngeal head and neck cancer [13,14].

## 3. The Epidemic of HPV-Associated Head and Neck Cancer and Trials of De-Escalated Therapy

Epidemiologic data suggesting a connection between HPV oral infection, sexual history, and cancer was emerging by the 1980s and early 1990s [15,16,17,18]. By the 1990s, epidemiologic data continued to mount; HPV type 16 (HPV16) seropositivity was described in head and neck cancer patients and HPV16 DNA was found in squamous cell carcinomas of the upper aerodigestive tract [19]. More extensive testing found that HPV16 was integrated, that p53 mutations occurred less frequently in HPV positive tumors, and that the oropharynx was the preferred site for development of these tumors [20]. Additionally, seropositivity against HPV16 was associated with a massive increase in risk of oropharyngeal cancer [21]. Early studies had conflicting results related to prognosis for HNSCC associated with HPV versus tobacco-associated tumors [22,23], but secondary analyses of a cooperative group trial settled the issue, clearly showing both an overall and a progression-free survival advantage for patients with HPV-positive tumors [24]. Retrospective analyses of this cooperative group study also categorized HPV-associated cancers into low or intermediate risk categories based on tobacco smoking history of greater than 10 pack-years and extent of neck metastases.

The risk categorization based on pack-years has been extensively adopted and continues to be used as a primary tool to identify patients at low risk appropriate for de-escalated therapy; however, emerging retrospective data from multiple groups would seem to indicate that tobacco exposure of 20–25 pack-years is a more accurate prognostic threshold [25]. Clinical trials have examined a variety of approaches for reducing therapy in HPV+ oropharynx cancer, with the use of chemotherapy to radically reduce radiation dose with a goal of diminishing late swallowing dysfunction, more modest reduction in radiation dose to reduce acute toxicity and duration of therapy, and transoral resection to permit pathology-driven treatment deintensification with the potential to spare chemotherapy and radiation. To date, strategies to de-escalate a specific modality have been driven by considerations of function and toxicity. As molecular characterization advances to predict sensitivity to radiation, cytotoxic chemotherapy, immunotherapy, or other targeted agents with continued development of biomarkers, patient selection and treatment paradigms will become more targeted and personalized.

The E1308 trial was an early study of de-escalated therapy for HPV-associated HNSCC. In this trial response to induction, chemotherapy was used as a predictor of radiosensitivity [26], with the goal of reducing radiation dose to the pharyngeal constrictors to below 56 Gy, a previously identified threshold associated with aspiration risk [27]. Here, patients with a clinical response to three cycles of chemotherapy received definitive cetuximab and radiation with radiation dose limited to 54 Gy. The overall 3-year survival in this trial was 85% [28], patients treated with reduced radiation dose had superior outcomes, and among nonsmokers with non-T4 and non-T3 cancers, no recurrences were observed, with a 3-year PFS of 96%. Late swallowing and oral pain were reduced in patients who received reduced radiation dose. These results have been confirmed in additional studies using alternative induction or concurrent systemic regimens [29,30].

Chera pioneered the use of a 60 Gy chemoradiation regimen for HPV+ oropharynx cancer, demonstrating an 86% pathologic complete response rate, and 3-year disease-specific survival of 100%, albeit the trial incorporated neck dissection [31]. NRG-HN002, a phase II randomized trial for low-risk, p16-positive, oropharynx cancer studied reduced-dose moderately accelerated radiation therapy (60 Gy over 5 weeks) with concurrent weekly cisplatin (40 mg/m^2^) [32]. The study sought to achieve at least 85% PFS and favorable swallowing outcomes. With a 2-year PFS of 90.5% (90% lower confidence bound, 86.6; *p* = 0.04), the 60 Gy concurrent regimen met these goals, but the radiation only arm failed. Concurrent chemotherapy plus 60 Gy, as in NRG-HN002, was advanced to be one arm of the three-arm randomized phase III trial, NRG-HN005, with the other arms being standard 70 Gy radiation delivered concurrently with high-dose cisplatin vs. 69 Gy radiation concurrent with the PD-1 inhibitor, nivolumab. Radiation fields in HN005 were reduced to preserve function and minimize immunodepletion of tumor-draining lymph nodes in the elective neck volume. Interestingly, the concurrent cisplatin and 60 Gy radiation arm of NRG-HN005 was halted on interim analysis due to a greater than anticipated number of treatment failures, although data on chemotherapy dose intensity and radiation fields have not been published to date. Concurrent chemoradiation with more drastic radiation dose reduction was studied in patients with normoxic tumors based on 18F-MISO imaging at 2 weeks into radiation treatment. Of 152 patients, 128 demonstrated normoxia after 2 weeks of therapy and received only 30 Gy radiation, with a 2-year PFS of 94% [33].

Among patients who are candidates for minimally invasive transoral surgery, deintensification of the postoperative treatment was studied in the ECOG-ACRIN trial, EA3311 [34]. Patients with T1 or T2 p16+ oropharyngeal tumors lacking evidence of extracapsular extension of nodal disease were included. All surgeons were credentialed for robotic surgery. Among 359 eligible patients, 38 had N0 or AJCC 7th edition N1 disease, and were observed, and 113 patients with high-risk disease because of involved margins, extranodal extension, or high node number received postoperative radiation to 66 Gy with weekly cisplatin. An additional 208 patients had intermediate risk disease defined as minimal or no extranodal extension and 2–4 involved nodes; these patients were randomized between 50 and 60 Gy postoperative radiation. Three-year PFS was 96.9% for low-risk patients, 94.9% for intermediate-risk patients treated with 50 Gy, 93.4% for intermediate-risk patients treated with 60 Gy, and 90.7% for high-risk patients. Of interest, in EA3311 and in contrast to trials of primary radiation, outcome was not worsened by tobacco history [35].

In sum, outstanding two- and three-year results can be achieved with deintensification schemes that reduce radiation with patient selection through induction chemotherapy, on treatment normoxia, or pathologic features following tumor excision and neck dissection. Basing patient selection for de-escalated therapy solely on clinical staging and smoking history has a varying history of success. Larger trials to compare these approaches to conventional chemoradiation and to improve patient selection criteria before treatment should be a priority for the field. The durability of the excellent early results of de-escalation trials should be carefully examined and updated as the data matures since tumor and functional outcomes at ten or even twenty years in these relatively young and healthy patients will be important.

## 4. HPV Carcinogenesis and Prognostic Biomarkers to Guide Therapy

Improved survival of patients with HPV-associated HNSCC compared to those with HPV-negative cancers combined with the morbidity and mortality associated with standard cisplatin-based chemoradiation therapy led to investigation of strategies to de-escalate therapeutic intensity, as described above. The major issue of determining how to select patients for de-escalation fell to TNM staging and smoking history, largely based on the retrospective analysis of the RTOG 1016 clinical trial [24]. An aspirational goal for all cancers has been personalization of therapy with accurate identification of patients who will have good outcomes to specific therapies. Failure of cetuximab to be as effective as cisplatin when given concomitantly with radiation further highlighted the need to identify patient cohorts likely to respond and rigorously test new therapies [36], but was also a blow for targeted therapies for de-escalation. The emergence of technological advancements including detection and quantification of circulating tumor HPV DNA (ctHPV-DNA), as well as improved molecular characterization of tumors, has led to new prognostic tools.

The potential clinical utility of plasma ctHPV-DNA has been demonstrated with excellent sensitivity and specificity for pretreatment diagnostic or post-treatment monitoring; see below for further discussion [37,38,39,40,41,42,43,44]. Studies from multiple groups have also explored dynamic changes in ctHPV-DNA during therapy, suggesting that these measurements may predict treatment outcome and guide the extent of therapy. Determining which kinetic measurement best correlates with outcome in patients treated with chemoradiotherapy is controversial, with some studies showing that it is rapid clearance of ctHPV-DNA [45], and other data suggesting that the best prognostic kinetic feature is an initial increase in plasma ctHPV-DNA [46]. For patients treated surgically, rapid clearance of ctHPV-DNA in the postoperative period was associated with better outcomes, as might be expected for a measure reflecting removal of disease [47]. Several studies have applied next-generation sequencing for determining plasma ctHPV-DNA, with the advantages of improved sensitivity over PCR-based approaches [44], while simultaneously providing information about HPV genotype and viral genomic integration into the host genome [48,49]. Considering that both integration status and HPV genotype have been correlated with outcome [50,51,52], assays providing multifaceted data may provide the most accurate prognostic information. These initial studies have identified treatment-related changes in ctHPV-DNA as a potential marker for treatment titration and suggest that patients with favorable kinetics could have radiation or chemotherapy doses reduced while those with unfavorable kinetics could complete standard therapy or undergo additional or novel therapy.

An interesting biological feature observed soon after acceptance of HPV as a cause of HNSCC was that a relatively high proportion of these tumors lacked HPV genome integration [53]. Several studies have estimated the percentage of integrated tumors using RNA expression data, exon capture, HPV capture, or whole-genome sequencing and found that 25–36% of HNSCC lack integration [54,55,56,57]. For virally integrated pharyngeal cancers, HPV integration is estimated to occur more than 25 years before diagnosis and there are significantly more integration sites per tumor when compared to uterine cervical cancer [57,58]. Preferential integration sites within or close to oncogenes and immune modulators were found to occur in multiple oropharyngeal tumor samples, and amplification and overexpression of genes near integration is common, suggesting that integration to disrupt these genes’ expression contributes to tumor formation or maintenance [54,57]. Sites within or near HPV insertions are also enriched in genes that could impact response to therapy [55]. Although unexplored as potential biomarkers of response, some examples of genes whose altered expression could impact response to therapy are the immune modifier CD274 (PD-L1), the EGFR family members ERBB2 (HER2) and HER3, and the DNA repair RAD51 homolog, RAD51B [54,55,56,59].

Absence of integration opposed the classical model of HPV oncogenesis developed through analysis of uterine cervical carcinoma. A consistent feature of this classical model is integration of the virus into the host genome to disrupt HPV E2 gene expression with resultant increased expression of HPV oncogenes E6 and E7. When considering head and neck cancers with HPV genome integration, direct comparison with uterine cervical cancers revealed that integration in HNSCC was less frequently associated with loss of E2 and more likely to occur in gene-rich areas with altered somatic gene expression [57]. HPV integration sites are frequently amplified and recurrently flank oncogenes MYC, SOX2, and p63 [54]. Integration sites are frequently clustered and home to fragile sites, transcriptionally active areas, and enhancer elements marked by FANCD2 and histone acetylation, and coaptation of enhancers with integration-associated amplification of the area to create super-enhancer-likes has been shown in uterine cervical cancer [54,60,61]. Interestingly, extrachromosomal circular DNA is frequently found in HPV+ HNSCC and may contribute to the creation of amplified super-enhancer areas as a driver of HPV gene expression [61,62].

The association of HPV integration with outcome in head and neck tumors has had varied and conflicting results, with some studies showing no correlation with survival [54,62], some a positive correlation with viral integration [56,63], and others an unfavorable correlation [55,64]. Interestingly, to our knowledge, all studies using transcriptional (e.g., RNAseq) methods to ascertain integration status have found integration to be associated with poor prognosis [55,64,65]. A recent multiomics study of uterine cervical cancer categorized integration sites as productive or silent based on the presence or absence of viral-human fusion transcripts [66]. Productive integration sites differed from silent sites in that they were more likely to have focal amplification, higher E6 and E7 expression, and disruption of E1 and E2. Tumors with productive integration presented at higher stage had less T cell and B cell activation signaling and were associated with more aggressive tumors. Among HNSCC with integration of HPV, 85% display viral-human chimeric transcripts with most initiated from viral promoters with disruption of somatic genes including a novel mechanism of NOTCH inactivation through disruption of MAML2 [54]. The concept of productive vs. silent HPV integration may further clarify prognostic implications of integration in head and neck cancer, especially in tumors harboring both integrated and episomal HPV.

The interaction of HPV-associated carcinomas with the immune response has also been intensively investigated and correlated with patient outcome. These efforts are not without solid footing, as seminal studies found that HPV-specific serum antibody positivity predicts improved prognosis in HPV-associated HNSCC [67]. Further investigation has revealed that HPV-specific B- and T-populations are frequently present in the microenvironment of these tumors [68,69]. Correlation of the tumor immune landscape as estimated by transcription profiles with survival revealed a three-gene signature that divided HPV+ HNSCC into three groups, designated as (1) immune-rich, (2) mixed, and (3) immune desert. The combined cohorts assigned roughly 1/3 of patients to each group, and survival analyses of several cohorts treated surgically or with standard or de-escalated chemoradiotherapy showed that patients with immune-rich tumors had improved disease-free (DFS) and overall survival compared to patients with immune desert or mixed tumors (see Table 1). Despite composite data showing significant differences in survival, comparison of immune-rich to mixed tumors in these cohorts showed wide confidence intervals with hazard ratios that frequently crossed 1. Since these cohorts were treated with different modalities and regimens, inability to accurately correlate with outcome could reflect that the classifier works better or worse, depending on treatment. Hazard ratios for individual cohorts were not provided for comparison of mixed tumors to immune desert tumors, but pooling all cohorts revealed a DFS hazard ratio that was not significant at 1.3; however, the hazard ratio for OS comparing these groups was significant, at 1.46 [70]. These analyses demonstrated that gross transcriptomic assessment of immune landscape correlates with patient outcomes across six distinct cohorts treated with either standard of care or investigational reduced intensity regimes.

Studies led by Sartor and colleagues represented groundbreaking attempts to sub-classify HPV+ HNSCC based on multiple correlated molecular features, including viral integration, viral gene expression, mutations and copy number alterations [71], and demonstrated correlation with outcome using such classifications [55]. Several groups identified that PIK3CA alterations in tumors were associated with poor survival [55,71,72]. These findings were extended to correlate PIK3CA defects with transcriptionally active viral integration sites and an immune-depleted microenvironment. Interestingly, this work also described that immune-rich, nonintegrated, low-risk HPV+ HNSCC tumors harbored a distinct association with copy number losses on chromosome 16 [71].

Our group has been intensively investigating a pathway-oriented approach, based on our observation that approximately 30% of HPV+ HNSCCs harbor destructive alterations (mutations or copy losses) in the NF-κB regulators, *TRAF3* and *CYLD*, the latter of which is on chromosome 16 [73]. More recently, we used an unbiased approach to identify prominent transcriptional profiles in these tumors based on coexpression of genes. Of the twenty-two profiles identified, only one intrinsically divided HPV+ HNSCC into subtypes based on high or low expression of genes defining the profile. Testing the profile on four independent cohorts revealed that patients whose tumors had high expression of profile genes had significantly improved survival compared to those with low expression of these genes (Table 1). Tumors assigned to the high expression vs. low expression subtypes were also distinguished by differences in somatic gene alterations, mutational signatures, HPV gene expression, HPV genome integration, and genomic methylation profiles [65]. The gene profile that identified the two subtypes of HPV+ HNSCC was based on high or low NF-κB signaling and fully recapitulated previous subtypes that our group defined based on the presence or absence of inactivating defects in *TRAF3* or *CYLD* or associated gene expression changes [64,65,73]. Based on high vs. low expression of genes in the NF-κB signature, roughly 50% of patients were assigned to each subtype and previously reported molecular markers of good or poor prognosis appropriately sorted with the two subtypes; specifically, estrogen receptor-alpha expression [74,75] segregated with the subtype having high NF-κB activity and good survival, and PIK3CA defects segregated with the subtype having low NF-κB activity and poor survival. An increased incidence of HPV genome integration, as determined by transcription of HPV genes or by presence of transcribed (nonsilent) integration sites, was found in the subtype identified by low NF-κB activity and associated with poor survival.

The identification of two subtypes of HPV-associated HNSCC has immediate utility for design of de-escalation trials. In addition to identifying tumors correlated with good outcome, our work showed that tumors with activated NF-κB signaling were more sensitive to radiation, implying that radiation, albeit at lower doses, may be a required part of de-escalation for this subtype. On the other hand, tumors in the subtype with low NF-κB activity are radioresistant, implying that they will require a standard therapeutic dose or that they may be better suited for surgical or alternative therapies. The use of smoking history to identify patients for deintensified therapy is questionable given that it miscategorized smokers in the good, NF-κB high, prognosis group, and that smoking history was not correlated with diminished survival for this subtype [65].

The pathway (NF-κB)-oriented approach is highly internally consistent, identifying distinctions in multiple molecular features of HPV+ HNSCC epithelial tumor cells (mutational processes, genomic methylation, viral integration, viral gene expression, frequently mutated genes, and ER-alpha expression). Tumor immune microenvironment differences were also noted between the subtypes, with the high NF-κB subtype associated with good prognosis having significantly elevated tumor infiltration of CD4+ regulatory T cells (Tregs), indicating that tumor cell NF-κB activation mechanistically relates to the immune microenvironment [65]. Examination of immune infiltration in cohorts containing both HPV+ and HPV-negative HNSCCs supports the association of higher CD4+ Treg cells in tumor and stromal compartments, with more favorable outcome [76]. Surprisingly, this study did not show significant survival advantage of HPV+ or p16+ tumors compared to HPV- or p16-negative tumors. Higher peripheral blood Treg level also correlated with improved overall survival in HPV+ HNSCC, with a trend toward improved disease-specific survival [77]. Further studies are needed to determine if there are other differences in tumor immune infiltration between HPV+ HNSCC subtypes.

## 5. Targeting of HPV+ HNSCC Molecular Vulnerabilities

As described above, clinical trials for patients with HPV-associated head and neck cancers are built on current and past experiences to safely advance therapeutic deintensification. An alternative strategy to improve care for these patients is to develop new targeted or less morbid therapies. Accompanying their development, markers are needed to identify patients whose tumors are less responsive to standard therapies or those that have molecular alterations that can be targeted. Improved understanding of HPV-associated head and neck cancers has highlighted many unique molecular characteristics of these tumors, some of which represent targetable molecular vulnerabilities.

Many current and planned de-escalation trials for HPV+ HNSCC focus on reducing radiation dose and/or field, but determinants of these tumors’ radiation sensitivity are only now emerging. In addition to its potential as a prognostic marker, finding that tumors in the high NF-κB activity subtype are more radiosensitive led us to find that NF-κB activation through loss of *TRAF3* or *CYLD* sensitized cultured cells to radiation [65]. The finding that HPV+ HNSCC cells with activated NF-κB were more sensitive to radiation was surprising and counterintuitive, given the large body of the literature implicating NF-κB activation in resistance to chemotherapy and radiation in many cancer types [78]. Although specific molecular mechanisms of how *TRAF3* or *CYLD* loss leading to constitutive activation of NF-κB sensitizes HPV+ HNSCC to radiation are still being investigated, our findings suggest that therapeutic NF-κB activation may increase radiation response in the subtype of these tumors harboring low NF-κB activity. We are currently exploring this hypothesis in preclinical models.

The Cancer Genome Atlas (TCGA) for head and neck cancer highlighted epigenetic differences between tobacco- and HPV-associated tumors [3]. Increased genome methylation was associated with HPV HNSCC and led our group to explore therapeutic effects of demethylation in these tumors. Demethylation markedly increased global transcription in HPV-associated tumor cells, but paradoxically decreased expression of HPV oncogenes E6 and E7 [79]. Decreased expression of E6 correlated with marked stabilization of p53 protein in cultured cells and in tumors from patients treated in a window trial. The window trial showed that demethylation therapy increased apoptosis only in HPV+ tumors, and experimental work suggested that cell toxicity was partially reliant on p53 activity. Further exploration of cytotoxic effects of demethylation found that it caused double-strand breaks that were dependent on transcription, translation, and the cytidine deaminase APOBEC3B (apolipoprotein B mRNA editing enzyme, catalytic subunit 3B) that is frequently overexpressed in HPV+ HNSCC [79,80,81].

In addition to deleterious effects of demethylation on HPV-associated head and neck cancer cells, demethylation also led to gene expression changes in these cells that could increase immune activation or recognition. Along with decreasing expression of HPV E6 and E7 genes, demethylation also inhibited expression of all early genes, including E5 [79]. HPV16 E5 is associated with resistance to immune checkpoint, anti-PD-L1, and therapy in head and neck cancer [82], where it has been shown to inhibit the immune response through decreasing expression of major histocompatibility complex (MHC) and inhibiting antigen presentation. High E5 expression in HPV+ HNSCC tumors correlated with worse survival, and pharmacological inhibition of E5 in preclinical models elicited a tumor response with increased MHC expression as well as T cell activation.

Additional immunostimulatory effects of demethylation in HPV+ tumors are related to increased expression of neoantigens. Using colon cancer as a model to investigate effects of demethylation, investigators found that demethylation activated viral mimicry in these cells through activating expression of endogenous retroviruses [83]. Given the high level of methylation seen in HPV+ HNSCC and massive transcriptional activation induced by demethylation, we are exploring demethylation-triggered viral mimicry as an additional vulnerability of these tumors. An additional antitumor effect of demethylation in HPV+ head and neck cancer cells is through increased expression of immune regulators and cytokines, including CCL5, interferon stimulated genes (ISGs), and interferon gene family members (IFITs). These immune modulators enhance immune response and attract T cells. Given the positive immune modulatory effects of demethylation observed in HPV+ HNSCC, including decreased E5 expression, potential for viral mimicry, and expression of immune modulators, combination of demethylation with anti-PD-1 therapy is currently being tested in a window clinical trial (PI Burtness, HIC #2000025632).

## 6. Biomarkers for Early Detection of Recurrent HPV+ Head and Neck Cancer

Blood tests to aid in cancer diagnosis or detection of recurrence are used for specific cancer types that overproduce tissue-specific markers or that commonly re-express proteins whose expression normally occurs only during fetal development. Oncologists and patients are familiar with many of these tests, including prostate-specific antigen (PSA), thyroglobulin (Tg), calcitonin, alpha-fetoprotein (AFP), carcinoembryonic antigen (CEA), and cancer antigen 125 (CA-125). Approved laboratory testing for expression of these cancer biomarkers requires sensitive testing methods, most commonly through development of specific antibodies. More recent sensitive techniques for detecting tumor-associated nucleotides or circulating tumor cells are revolutionizing biomarker development for all tumor types.

The expanded promise of liquid biopsy to advance cancer care Is indicated by its inclusion in more than 500 clinical trials in the NCI portfolio [84]. The liquid biopsy approach for detection of unknown cancers in high-risk individuals has relied on detection of common mutations or methylation patterns in circulating DNA to assign risk. For a screening test to be used on the general population, challenges remain, since both sensitivity and specificity must be extremely high. Sensitivity is challenged by very small cancers that may shed DNA that is below the limit of detection, while inadequate specificity may lead to large numbers of false positives in low-risk populations. Accuracy for cancer detection can be especially challenging for liquid biopsies dependent on cancer-associated mutations, because aging is associated with both higher cancer risk and increased background mutations of unknown significance, most of which arise from clonal hematopoiesis [85].

In addition to its applications in early detection of unknown cancers, liquid biopsy has potential utility for monitoring therapy and staging. Evidence continues to accumulate in lung and colon cancer, supporting the use of liquid biopsy for early detection of tumor recurrence with the goal of identifying the recurrence when it is still possible to effectively treat it [86,87]. Many challenges of liquid biopsy associated with screening low-risk individuals for asymptomatic cancers are circumvented when liquid biopsy is used for early detection of recurrences, since exact mutations identified in the original tumor can be targeted for detection in circulating tumor DNA. While small volume of recurrent tumor may limit how early recurrences can be identified, studies suggest that detection of circulating tumor cells and circulating tumor DNA precedes detection of recurrence by standard follow-up [88]. The detection of recurrences before they can be localized by imaging or physical exam can lead to treatment dilemmas and patient anxiety in the absence of treatment options. If a targetable lesion cannot be identified, local treatments such as radiation and surgery are not possible, leaving the options of waiting for appearance of targetable lesions or systemic therapy.

Circulating tumor cells (CTCs), both before treatment and their persistence after therapy, correlate with poor survival in breast, colon, and prostate cancer [89]. In HPV+ HNSCC, CTCs were correlated with advanced nodal classification, being more frequently found in N2B or higher (AJCC 7th edition), and had a nonsignificant trend with increasing tumor volume, but not T classification [90,91]. Detection of CTCs is technique-dependent, and as methods for their detection have advanced, circulating tumor cells have been explored in earlier-stage tumors, including HPV-associated head and neck cancer [92]. Although the cell rolling and dendrimer-conjugated antibodies increased sensitivity with CTCs detected in 13 of 14 intermediate-stage HPV+ cancers, there was no correlation of recurrence with treatment-related changes in CTCs. Although HPV status was not directly queried, oropharyngeal tumors had a trend of improved overall and disease-free survival when CTCs were detected, as opposed to HPV-negative HNSCC, which followed the accepted trend where CTC detection was associated with poor survival [91].

Although circulating tumor cells, RNA, exosomes, and metabolic and immune profiles are exciting applications of liquid biopsy and are being tested for detection of recurrent disease or response to therapy, analysis of circulating tumor DNA (ctDNA) is most advanced in HPV+ HNSCC [84]. ctDNA detection for HPV+ HNSCC has several advantages, given that the tumors are addicted to HPV E6 and E7 oncogenes and must maintain their expression; therefore, head and neck cancer cells must maintain all or part of the viral genome [93]. In HNSCC, the physical state by which HPV DNA is maintained correlates with different mechanisms of HPV carcinogenesis [65]. HPV DNA can be integrated into the host genome, maintained as extrachromosomal DNA, or found in both integrated and extrachromosomal forms [57,62]. In addition to losing or disrupting expression of the HPV E2 gene, integration is frequently associated with HPV DNA amplification, and tumors lacking HPV integration replicate the HPV episome, leaving each cell with multiple copies of truncated or full-length HPV sequences [62,94]. Recent studies have also shown that HPV DNA can also be combined with somatic DNA and hybrid human/HPV sequences maintained as amplified extrachromosomal constructs [62].

Regardless of integration status, HPV+ HNSCC cells contain multiple copies of the HPV genome, increasing the likelihood of detecting even small recurrent tumors through ctHPV-DNA, as several copies of the biomarker are present in each tumor cell. An additional advantage of liquid biopsy for detection of recurrent HPV+ HNSCC is that oral infection with HPV is not detected in plasma, suggesting that only cancers shed HPV into the blood [95]. Regardless of the carcinogenic pathway employed, required retention of HPV DNA sequences in tumor cells and absence of HPV shedding by noncancer oral infections makes its detection in circulation extremely specific, and amplification of HPV DNA in HNSCC markedly improves sensitivity.

The potential for clinical utility of circulating tumor HPV DNA for detection of recurrent HPV-associated HNSCC has been shown in retrospective studies from individual or consortia of institutions. The head and neck oncology group from the University of North Carolina used digital PCR to analyze plasma of patients with HPV+ HNSCC after standard or de-escalated therapy [38]. Of 115 post-treatment patients, sixteen had circulating HPV DNA detected in two consecutive samples, and of these, fifteen developed recurrences for a positive predictive value of 94%, with a lead time before biopsy-proven disease of 3.9 months. A Copenhagen University study also used digital droplet PCR for detection of circulating tumor HPV DNA in post-treatment HPV+ HNSCC [43]. Analyses of samples from 54 patients with a cutoff of a single positive result had a positive predictive value of 100% and negative predictive value of 71% for detection of recurrent or persistent disease. These results have led many clinicians to use circulating HPV DNA as a complement to office endoscopy and imaging for following patients with HPV+ HNSCC after therapy. A study from Japan used digital droplet PCR analysis of plasma from treated HPV+ HNSCC patients to compare circulating tumor HPV DNA testing with CT-PET [96]. Thirty-five patients were followed for 21 months with ctHPV-DNA and CT-PET. Comparison of test performance yielded similar negative predictive values for the two modalities while the positive predictive value of ctHPV-DNA was much higher than CT-PET, 100% vs. 50%. A study from eight institutions and the Naveris corporation retrospectively evaluated 543 patients after treatment of p16 positive HNSCC, as a surrogate marker for HPV [42]. This study evaluated performance for detection of recurrence of the Naveris test for ctHPV-DNA with standard imaging. In agreement with the Japanese results, this study also found that NPVs were similar for the two modalities, 99% vs. 98%, while PPV was higher for circulating HPV DNA vs. imaging, 95% vs. 75%. A positive circulating tumor HPV DNA test preceded confirmation of recurrence by 59 days in this study, which also found that recurrence-free and overall survival were markedly worse for patients with a positive liquid biopsy, HRs 22.5 and 8.0, respectively. These promising retrospective results led to an ongoing multi-institutional prospective biomarker study sponsored by the NIDCR (U01DE029754), testing the efficacy of ctHPV-DNA to detect recurrences in patients following definitive treatment of HPV+ HNSCC.

## 7. Conclusions

Biomarkers for HPV+ HNSCC have been identified and are moving into clinical care to guide therapy. Understanding of molecular vulnerabilities associated with HPV carcinogenesis has led to new therapeutic targets that are being tested preclinically and in clinical trials. Perhaps the most advanced biomarkers are circulating HPV DNA for early detection of recurrence and tumor intrinsic gene defects or gene expression to identify HPV+ HNSCCs with enhanced response to radiation and those that are associated with improved survival. Early detection of recurrence may offer more patients curative salvage therapy, and identification of tumors that are more responsive to therapy has the potential to advance personalized cancer care by identifying those patients most likely to be cured with de-escalated therapy.

## Figures and Tables

**Table 1:** Independent cohorts, patient numbers, p values and hazard ratios for prognostic expression signatures in HPV+ HNSCC.

**Prognostic Gene Expression Signature**	**# of Transcripts** **In Signature**	**Name and % of Patients in Subtypes Defined by Signature**	**Independent Cohorts**	**# of Patients in Cohorts**			
**NF-κB**	203	**NF-κB high (49%)** **NF- κB low (51%)**			**NF-kB high vs low**		
					***p* value**	**HR PFS (95% CI)**	
			UNC	104	0.02	0.27 (0.09–0.84)	
			TCGA	61	0.02	0.09 (0.01–0.95)	
			Vanderbilt	93	0.03	0.35 (0.13–0.96)	
			E1308	59	0.02	0.27 (0.09–0.87)	
**UWO3** **(immune)**	3	**Immune Rich (32%)** **Mixed (37%)** **Immune Desert (31%)**			**Immune Desert vs** **Immune Rich**		**Mixed vs** Immune Rich * **HR PFS (95% CI)**
					***p* value**	**HR PFS (95% CI)**	
			TCGA	71	0.01	6.18 (1.23–31.08)	8.32 (0.13–537)
			JHU	47	0.02	24.42 (0.85–704.38)	6165 (2.10–1.819 × 10^7^)
			LHSC	43	0.003	18.89 (2.41–148.25)	45.7 (0.29–7079)
			TMA	197	0.038	3.73 (1.1–12.61)	9.12 (0.51–162)
			WashU/Vandy	262	0.01	2.48 (1.33–4.65)	3.72 (0.74–19.05)
			BD2Decide	286	0.03	2.49 (1.17–5.33)	6.46 (1.26–33.11)

* *p* values not provided for this comparison; HR—hazard ratio; PFS—progression free survival.

## Data Availability

Not applicable.
